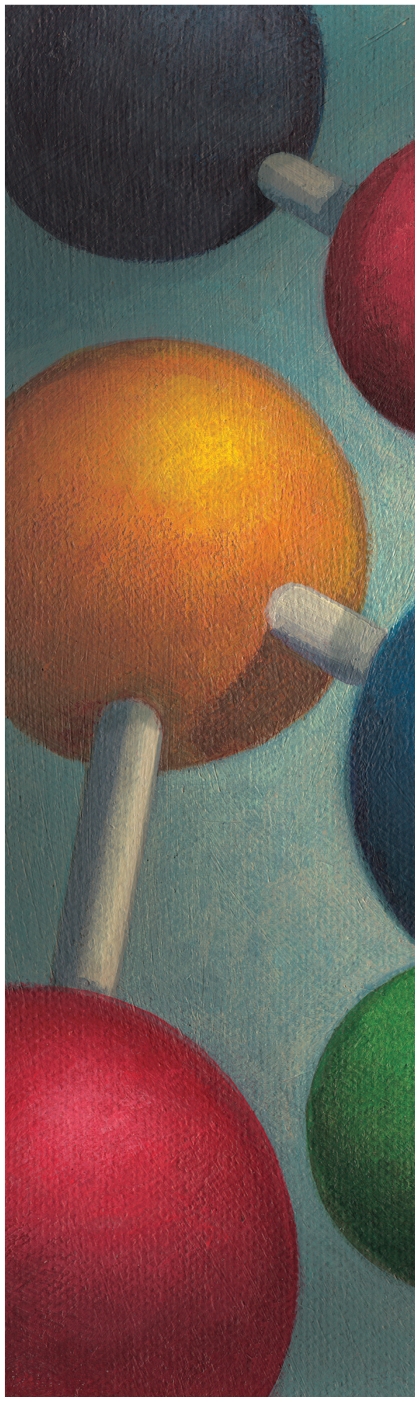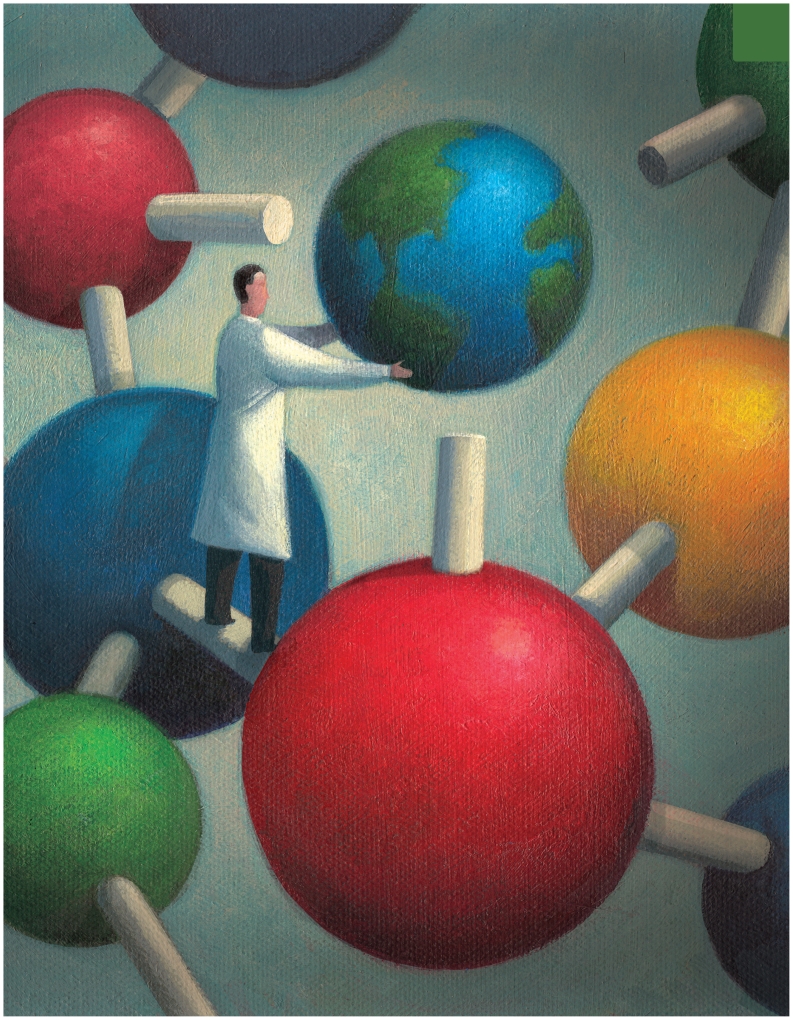# Principles into Practice Setting the Bar for Green Chemistry

**DOI:** 10.1289/ehp.118-a254

**Published:** 2010-06

**Authors:** David A. Taylor

**Affiliations:** **David A. Taylor** writes for *The Washington Post* and *Smith-sonian* and is author of *Ginseng, the Divine Root*, about the science and subculture surrounding the medicinal plant. He teaches science writing at The Writer’s Center in Maryland

Recent years have seen a disheartening string of revelations in which everyday items once considered safe—food packaging, toys, clothes, furniture, electronic components, and many more products—are found to contain carcinogens, endocrine disruptors, and other harmful chemicals.^1^ Growing demand for healthier alternatives, already seen in food production and housing construction,^2^ is also happening at the building-block level of manufacturing, where so-called green chemistry represents a revolutionary change in preventing pollution and health problems starting at the chemical design stage. Many industry and government entities are beginning to espouse the principles of green chemistry on their websites and in public statements. Now comes the task of crafting policy to put those principles into action.

The U.S. Environmental Protection Agency (EPA) defines green chemistry as “the design of chemical products and processes that reduce or eliminate the use or generation of hazardous substances. Green chemistry applies across the life cycle of a chemical product, including its design, manufacture, and use.”^3^ Green chemistry also aims to mitigate the type of uncertainty Alan Gold-berg, a professor of toxicology at the Johns Hopkins Bloomberg School of Public Health, recently described to *The New York Times*: “I can get [toxicity] information on only 20 percent of chemicals we interact with on a daily basis.”^4^ Of that 20%, he now says, he may be able to find information on overt toxicity for about half, but for details on specific effects such as developmental neurotoxicity, the figure shrinks toward zero.

So what does green chemistry look like? Consider the example of pregabalin, the active ingredient in the neuropathic pain drug Lyrica^®^. Pfizer developed an alternative green-chemistry process that converted several steps of pregabalin synthesis from use of organic solvents to water. That reduced both health hazards and production heating requirements. With the new synthesis, waste from the process dropped from 86 kg of waste per kg product to 17 kg, and energy use dropped by 82%.^5^

Proponents say that’s how the field can offer a win–win–win solution: good performance, lower cost, and less environmental impact—what Richard Engler, program manager of the EPA Green Chemistry Program, calls the “triple bottom line.”

For many, a standard is a logical next step. “At some point you have to go beyond a definition and principles,” says Engler. “I think that’s something the standard will enable.”

## Crafting a Standard

The American Chemical Society (ACS), a congressionally chartered independent professional organization, through its Green Chemistry Institute^®^ (GCI), is working with federal agencies, nonprofits, end users, and industry to craft a “business-to-business”^6^ standard for measurably reducing hazardous materials in all kinds of products and processes. The effort, says ACS GCI director Robert Peoples, is very close to having a standard to be instituted by the American National Standards Institute (ANSI), a nonprofit organization that has coordinated voluntary industry standards in the U.S. private sector since 1918. Peoples says the ACS aims to issue its draft for public comment by this summer.

Peoples compares the likely impact of an ANSI standard to that of LEED^®^ (Leadership in Energy and Environmental Design), the professional standard for green construction for which the U.S. Green Building Council offers accreditation. An ANSI standard would motivate whole industries to reconfigure their production processes for lower environmental and health impacts, says Jim Solyst, a principal consultant with ENVIRON International Corporation.

Working in partnership with the standards development organization NSF International, ACS GCI established a Joint Committee, which has been guided by two subcommittees and a balance of industry, nongovernmental, public health, academic, and government representatives. The standard-development process and meetings are open to anyone interested, says Peoples. He anticipates ACS GCI will roll out a finalized standard by late fall 2010, adding, “That’s going from a blank sheet of paper to an ANSI standard in just under two years.”

Like LEED, the ANSI standard will be voluntary. Companies will be able to confirm their adherence through one of several likely certification levels. The most stringent is independent third-party certification; below that is certification by a trade association or other party separate from the company. A voluntary standard, says Peoples, acknowledges that finding new ways of formulating many items won’t happen overnight. “Voluntary standards allow the marketplace to drive the changes. It will take a long time, and there’s no single path or approach,” he says. By setting a high bar, however, a standard drives everyone to a higher level of performance, Peoples says.

The EPA has been a key federal agency partner in developing a standard since green chemistry was “born” there in the early 1990s (the current EPA assistant administrator for research and development, Paul Anastas, is one of the authors of the 12 principles of green chemistry^7^). Others involved include the U.S. Department of Commerce, which has developed its Sustainable Manufacturing Initiative to support the implementation of sustainable manufacturing practices and explore its implications for U.S. global competitiveness and firm profitability, says Blandine Trouille, a senior trade analyst with the department.

Trouille notes that the September 2009 meeting of the G-20 (leaders from 20 countries that together represent 85% of the world’s economy) “was all about sustainable economic development. That means manufacturing in a different way.” With that emphasis in the global marketplace, she adds, “Having a standard helps an industry or company show they’re not simply talking the talk, but walking the walk.” And other stakeholders acknowledge that since information on environmental health problems is now more accessible, the marketplace itself demands solutions faster than ever before.

## Greening the Defense Industry

Department of Defense (DOD) officials expect their own guidelines and policies, along with broader federal ones, will have more impact than an ANSI standard. The DOD wrestles with the particular needs of its national security mission, which can make chemical substitutions difficult, says Carole LeBlanc, special expert on chemical and material risk management in the DOD Office of the Deputy Under Secretary for Installations and Environment. Those needs include long-lived products, critical performance standards with little flexibility, and high-volume production, says LeBlanc, who leads the department’s effort in green chemistry. “The mission of national security always wins in a tie [with other objectives],” says LeBlanc. But green chemistry principles can still come into play.

For example, trichloroethylene (TCE), an organic solvent, is still used in many applications although it’s a likely human carcinogen.^8^ When DOD staff proposed replacing TCE with methylene chloride, another solvent, LeBlanc weighed the options with an eye to uncertainty as well as known risk, recognizing that regulations lag behind scientific knowledge. Methylene chloride may affect the nervous system but its role in human cancers is still inconclusive.^9^ In that instance, the DOD deferred replacing TCE. Explains LeBlanc: “We know exactly what TCE is going to do”—and therefore what precautions to take; the precautions for methylene chloride are less understood.

LeBlanc cites an earlier EPA program, the Significant New Alternatives Policy (SNAP) Program, as a model policy approach that identified best practices for selecting chemicals. SNAP prioritized problems and solutions: it first identified the easiest fixes and mandated their use, then identified harder issues for the medium and long term. “That set a road map for green chemistry,” she says.

LeBlanc’s office works through two channels: shaping policy through directives and instructions and through less formal interactions with the branches of the armed services. “The policy piece drives where the road and the rubber meet,” she explains, but there are also “wonderful things you can achieve rather informally” through workshops across the service branches that reveal shared problems and possible solutions.

LeBlanc and colleagues identified best management practices for green chemistry that already existed at DOD. From there, they developed training curricula on acquisition systems for online courses at the Defense Acquisition University, which trains 126,000 DOD staff in acquisition, technology, and logistics. Those courses are in the final stages of review. “When you change a chemical, you almost always have to change a process,” explains LeBlanc, and that requires training in the new process.

Also, after reviewing its existing programs for assessing chemical composition and safety, the DOD adopted a longer-term approach for tracking chemicals used by military personnel and contractors. A life-cycle assessment approach now examines products and chemicals through three phases of their use: acquisition and procurement, maintenance (the phase when most chemical exposures occur, says LeBlanc), and disposal. “This is a whole new way of looking at things for DOD,” says LeBlanc. A June 2009 DOD workshop on reducing hazardous materials also supported the military branches in identifying problem chemicals and planning solutions. Since then, two similar workshops have taken place at the National Aeronautics and Space Administration and the Aerospace Industries of America, a contractors’ group.

## Carrots and Sticks

Among policies that promote green chemistry, the 2006 legislation by the European Union known as REACH (Registration, Evaluation, Authorisation and Restriction of Chemical Substances) is the most imposing. It addresses the human health and environmental impacts of all chemicals, mandates comprehensive information, and will be phased in over a decade. REACH will require companies to register chemicals they use with a new agency based in Finland. REACH applies to all chemicals produced in or imported into Europe.

It’s too early to determine that law’s health impact, but its impact on policy is already formidable. “REACH mandated the provision of information,” explains Solyst, “a vast increase in the amount and availability of information on chemicals. That’s good. And it has made downstream users of chemicals much more aware.” Before, he says, there was not sufficiently detailed information to make the public confident of the origin and composition of many chemicals used in daily life. REACH provides that level of information—but also creates a huge task in managing it.

For LeBlanc, REACH is in part an attempt to give Europe a competitive advantage in a global shift toward sustainability. “This is very much a trade issue for the United States,” she says, adding it will have “a huge impact” on the DOD’s supply chain and information in its Material Safety Data Sheets.

Stateside, federal governmentwide programs for greening procurement include the EPA’s Environmentally Preferable Purchasing and the Green Procurement Program managed by the General Services Administration. One key incentive program the EPA manages is the Presidential Green Chemistry Challenge Awards, which began in 1996 during the Clinton administration. The awards are typically given annually to outstanding individuals and businesses responsible for innovations in green chemistry in five categories: academic research, small business, designing greener chemicals, greener synthetic pathways, and greener reaction conditions. The awards have raised awareness of green chemistry and fostered the idea that with innovation, you can reduce both environmental impact and cost, Engler says; he explains, “It’s not a tradeoff between the two.”

Others are less sure. Elizabeth Gross-man, an investigative journalist and author of *Chasing Molecules: Poisonous Products, Human Health, and the Promise of Green Chemistry*,^10^ sees mixed motives in the chemical industry. “Every large chemical company is investing in green chemistry,” she says. “At the same time, those companies have huge long-term investments in the chemicals being substituted—for example, phthalates—and their shift off the market will put a dent in the revenue from longstanding products.”

Engler and LeBlanc agree there are no drop-in replacements for any toxic chemical used commercially. Green chemistry proceeds application by application, looking for potential substitutes based on desired functions.

Meanwhile, congressional reform of the 1976 U.S. Toxic Substances Control Act (TSCA) is on its way,^10^ and individual states are taking action to protect citizens against chemicals shown to cause adverse health effects. California, for example, has banned the sale of toys and other children’s items that contain certain phthalates and sets the strictest standard in the United States for phthalates allowed in consumer goods.

Peoples notes the California initiative “is not about green chemistry per se, but is focused on regulation and information disclosure. Green chemistry as envisioned by the creators, is about reducing or eliminating the use of hazardous materials. It is not about sharing information about hazardous materials. There is a very big difference.”

He adds, “Our hope is that at some point in the future all chemistry will simply be green chemistry. That day is generations in the future, but the journey has begun.”

## The 12 Principles of Green Chemistry^7^

1. Prevention: It is better to prevent waste than to treat or clean up waste after it has been created.
2. Atom Economy: Synthetic methods should be designed to maximize the incorporation of all materials used in the process into the final product.
3. Less Hazardous Chemical Syntheses: Wherever practicable, synthetic methods should be designed to use and generate substances that possess little or no toxicity to human health and the environment.
4. Designing Safer Chemicals: Chemical products should be designed to effect their desired function while minimizing their toxicity.
5. Safer Solvents and Auxiliaries: The use of auxiliary substances (e.g., solvents, separation agents, etc.) should be made unnecessary wherever possible and innocuous when used.
6. Design for Energy Efficiency: Energy requirements of chemical processes should be recognized for their environmental and economic impacts and should be minimized. If possible, synthetic methods should be conducted at ambient temperature and pressure.
7. Use of Renewable Feedstocks: A raw material or feedstock should be renewable rather than depleting whenever technically and economically practicable.
8. Reduce Derivatives: Unnecessary derivatization (use of blocking groups, protection/deprotection, temporary modification of physical/chemical processes) should be minimized or avoided if possible, because such steps require additional reagents and can generate waste.
9. Catalysis: Catalytic reagents (as selective as possible) are superior to stoichiometric reagents.
10. Design for Degradation: Chemical products should be designed so that at the end of their function they break down into innocuous degradation products and do not persist in the environment.
11. Real-Time Analysis for Pollution Prevention: Analytical methodologies need to be further developed to allow for real-time, in-process monitoring and control prior to the formation of hazardous substances.
12. Inherently Safer Chemistry for Accident Prevention: Substances and the form of a substance used in a chemical process should be chosen to minimize the potential for chemical accidents, including releases, explosions, and fires.

## Figures and Tables

**Figure:**